# Supplementary figures and images for: Classification of commercial districts based on predicting the survival rate of food service market in Seoul
Source: PLoS One. 2025 Jul 10;20(7):e0326307. doi: 10.1371/journal.pone.0326307 (PMC12244476; doi:10.1371/journal.pone.0326307)

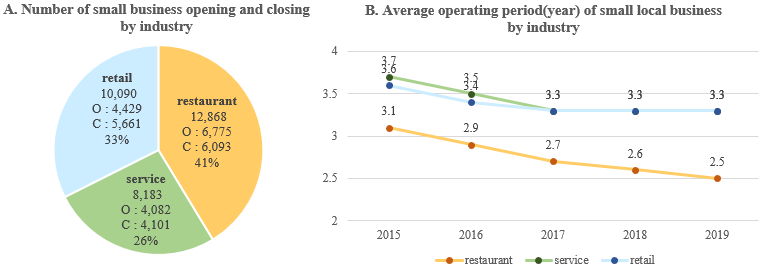

Supplement: S1 Fig — (TIF) [file pone.0326307.s001.tif]
