# Supplementary material for: Classification of commercial districts based on predicting the survival rate of food service market in Seoul
Source: PLoS One. 2025 Jul 10;20(7):e0326307. doi: 10.1371/journal.pone.0326307 (PMC12244476; doi:10.1371/journal.pone.0326307)
Supplement: Attachment — Submitted filename: Response to Reviewers.docx [file pone.0326307.s003.docx]

**Responses to Editor**

Thank you for your valuable comments. The recommendations and comments definitely improved the quality of this paper. Below are the major changes made during the revision process.

1. Please ensure that your manuscript meets PLOS ONE's style requirements, including those for file naming. The PLOS ONE style templates can be found at

https://journals.plos.org/plosone/s/file?id=wjVg/PLOSOne_formatting_sample_main_body.pdf and

https://journals.plos.org/plosone/s/file?id=ba62/PLOSOne_formatting_sample_title_authors_affiliations.pdf

We have reviewed and incorporated the PLOS ONE style requirements into our manuscript, including file naming conventions. I appreciate your suggestion.

2. Please note that PLOS ONE has specific guidelines on code sharing for submissions in which author-generated code underpins the findings in the manuscript. In these cases, we expect all author-generated code to be made available without restrictions upon publication of the work. Please review our guidelines at https://journals.plos.org/plosone/s/materials-and-software-sharing#loc-sharing-code and ensure that your code is shared in a way that follows best practice and facilitates reproducibility and reuse.

3. We note that you have indicated that there are restrictions to data sharing for this study. PLOS only allows data to be available upon request if there are legal or ethical restrictions on sharing data publicly. For more information on unacceptable data access restrictions, please see http://journals.plos.org/plosone/s/data-availability#loc-unacceptable-data-access-restrictions.

Data security concerns in this study can restrict data sharing. The data and code contain personally identifiable information, which the Seoul Credit Guarantee Foundation under the Seoul Metropolitan Government restricts public sharing. We might be able to provide the data and code to confirm the data filtering processes, but it cannot be shared publicly for any purpose.

Requests for data access can be sent to movementhyun@seoulshinbo.co.kr.

4. We note that Figures 2, 5, 6, 7, 9 and 10 in your submission contain map/satellite images which may be copyrighted. All PLOS content is published under the Creative Commons Attribution License (CC BY 4.0), which means that the manuscript, images, and Supporting Information files will be freely available online, and any third party is permitted to access, download, copy, distribute, and use these materials in any way, even commercially, with proper attribution. For these reasons, we cannot publish previously copyrighted maps or satellite images created using proprietary data, such as Google software (Google Maps, Street View, and Earth). For more information, see our copyright guidelines: http://journals.plos.org/plosone/s/licenses-and-copyright.

Figures 2, 5, 6, 7, and 9 were created by the authors specifically for this manuscript and do not involve any copyright issues. We used the ESRI base map for Figure 10. Since it is confirmed that the map can be used in academic publications, we believe that there would be no copyright issue to use the map in the manuscript. The link below provides detailed information on the terms of use for static maps for ESRI.

<https://doc.arcgis.com/en/arcgis-online/reference/static-maps.htm>

1. In the online submission form, you indicated that your data is available only on request from a third party. Please note that your Data Availability Statement is currently missing [the name of the third party contact or institution / contact details for the third party, such as an email address or a link to where data requests can be made]. Please update your statement with the missing information.

We updated the Data Availability Statement in the online submission form as requested. I appreciate your suggestion.

2. Please remove your figures/ from within your manuscript file, leaving only the individual TIFF/EPS image files.  These will be automatically included in the reviewer’s PDF

We removed the figures from the manuscript and uploaded all images separately in TIFF format as requested. I appreciate your suggestion.

3. Thank you for clarifying these points. Please clarify what program(s) were used to create the maps in Figures 2, 5, 6, 7, and 9

We used ArcGIS Pro 10.4 to create the maps in Figures 2, 5, 6, 7, and 9.

**Responses to Reviewer 1**

Thank you for your valuable comments. The recommendations and comments definitely improved the quality of this paper. Below are the major changes made during the revision process.

Your research on the chronic closure of small businesses in South Korea addresses a critical economic issue with significant implications for policy and economic stability. The use of a large dataset of 3,000,000 individual commercial facility data spanning from 2004 to 2018 provides a robust foundation for your analysis. The dual focus of your study—developing a deep-learning based model to simulate survival rate patterns and reclassifying commercial districts based on prediction outcomes—is both innovative and highly relevant.

Your application of the Long Short-Term Memory (LSTM) model to predict and simulate the survival rate of commercial facilities is particularly noteworthy. The results indicating future decreases in survival rates in CBD areas, contrasted with increases in university districts and IT industry clusters, offer valuable insights. These findings not only enhance our understanding of commercial dynamics but also provide quantitative evidence that can inform more targeted and effective policy interventions.

The author’s answer

Thank you!

1. Figure 1 fits in the appendix or this figure is not needed.

The author’s answer

We moved Figure 1 to the Supporting Information as recommended. Please see S1 Fig. I appreciate your suggestion.

2. More literature citations are needed. For example, lines 57 through 61 need to be supported by official reports or literature data.

The author’s answer

We added additional references to support the statements in lines 57 through 61. Please see line 61-65. I appreciate your suggestion.

3. The “Year” in Table 3 means that the article is a cross-section?

The author’s answer

We revised the “Year” in Table 3 and 4 to “Time Interval” and updated 2004, 2009, and 2014 to 2004–2008, 2009–2013, and 2014–2018, respectively. Please see Table 3 and 4. I appreciate your suggestion.

4. A note needs to be added below each image and table.

The author’s answer

We double-checked every figure and table and added additional information, such as legends and scale, where necessary. Please let us know if we have misunderstood your suggestion.

5. The article is documented by year, so the major events mentioned at the end of the manuscript need to be analyzed as a split.

The author’s answer

We inserted a section explaining the significant economic events that occurred during the study period. Please see line 43-48. I appreciate your suggestion.

6. The first letter of 4.1.2 needs to be capitalized.

The author’s answer

We capitalized the first letter of 4.1.2 as suggested. Please see line 314. I appreciate your suggestion.

7. However, it would be beneficial to expand on the specific variables and features used in the LSTM model. Additionally, discussing the potential limitations of your model and the generalizability of your findings beyond Seoul could provide a more comprehensive view of your research.

The author’s answer

As the reviewer pointed, we considered but could not include variables such as industry type, number of employees, region, and economic growth rate due to the model limited specifically to the Seoul city and data limitations. Including these variables in future research could enhance the generalizability of the model. We inserted a section explaining the variables considered but not included in the analysis as a potential limitation of this study, according to the reviewer suggestion. Please see line 179-184. I appreciate your suggestion.

**Responses to Reviewer 2**

Thank you for your valuable comments. The recommendations and comments definitely improved the quality of this paper. Below are the major changes made during the revision process.

The paper presented a very interesting topic analyzing the business survival rate in conjunction of the district wise business activity. It provided insightful conclusions regarding the business pattern within Seoul, and presented a generalizable framework and methodologies to gain valuable insights into local small businesses within major cities. Given the comprehensive techniques applied, well elaborated insights and good presentation, I recommend the paper to be published.

The author’s answer

Thank you!

1. (1) Could we provide more information regarding data normalization and cleaning. (2) The authors mentioned using industry wise data to create industry specific index and diversity index, is there any normalization performed between these industry level data, making sure that each industry data is at a similar level, with no outlier industry(s). The selection of the industries seem to have been provided in ref [23] and [28], it would be great if the authors could also provide the list of the industries used in the experiment within the paper for a full comprehensive understanding.

The author’s answer

(1) We did not apply normalization or standardization to the data used for training the LSTM model. There are two potential approaches for normalizing the data: first, normalizing across the group of 4,034 pixels for each time interval, and second, normalizing across the three-time intervals for each pixel. The first approach would replace the pixel values with relative values compared to other pixels within Seoul, which would make it challenging to reflect absolute changes when analyzing the data over time. On the other hand, the second approach is limited to having only three-time intervals for each pixel, which results in a group size too small for effective normalization. In this study, the model achieved a high level of accuracy despite not applying normalization. However, if more extended time-series data becomes available, normalization processes, as suggested by the reviewer, could further improve the accuracy of the model results.
(2) As the reviewer suggested, we added an explanation of the industry classification used in this study. Please see line 190-194. I appreciate your suggestion.

2. Authors created models based on constructed data within three time intervals, each of 5 years. Could we elaborate more regarding how the cutoff years were selected, and why did we choose to predict a 5 year survival rate instead of a different X year survival rate?

The author’s answer

In South Korea, the average operational period for small businesses is 5.6 years, and most studies has also used the 5-year survival rate to track the business performance of small enterprises. We added an explanation of why the cutoff years were set to 5 years for survival rate prediction in this study. Please see line 170-175. I appreciate your suggestion.

3. It would provide a more complete picture regarding how the LSTM model was trained, if we could provide examples of input data. LSTM is more superior in making predictions relying on data with sequential patterns, thus, a natural question would be, are we predicting survival rate using time series data or are we just using each independent variable combined? From here, providing examples of input data could provide clear insights into such questions.

The author’s answer

As the reviewer suggested, we added details about how the LSTM model was trained. For better understanding, we created a conceptual diagram showing LSTM based prediction processes. Please see line 219-223 and Fig 4. I appreciate your suggestion.

4. Why did we choose to form the problem as a regression problem, since we are predicting a probability? What would be the argument between forming the problem as a classification, i.e. predicting survival or not, vs. forming the problem as a regression one? Is there any concern regarding the bounding values of predicted dependent variables, including probabilities larger than 1 or less than 0? If there is such concern, have we performed steps to normalize the outputs?

The author’s answer

In this study, we predicted the 5-year survival rates for each pixel from 2019 to 2023. Since each pixel contains multiple businesses, we concluded that predicting survival probabilities as continuous values between 0 and 1 would better capture the variation between pixels than using a classification approach to predict binary outcomes (0 or 1). This approach avoids reducing important details, such as the differences between pixels. Similarly, widely used survival analysis techniques, such as Kaplan-Meier Estimation, calculate survival rates as continuous values to provide more accurate and detailed estimates.

All predicted survival rates in this study fell within the range of 0 to 1, so there was no need to apply normalization or boundary adjustment processes to the results.

**Responses to Reviewer 3**

Thank you for your valuable comments. The recommendations and comments definitely improved the quality of this paper. Below are the major changes made during the revision process.

The manuscript presents a valuable attempt to classify commercial districts in Seoul based on the survival rates of food service businesses using an LSTM-based prediction model. However, the paper requires revisions in methodology. Below are my detailed comments and suggestions for improvement.

The author’s answer

Thank you!

1. While the study addresses an important issue, the novelty of the approach is questionable. The use of LSTM models for time-series prediction is well-established, and the application to commercial districts, while interesting, does not seem to provide significant new insights or methods.

The author’s answer

The key novelty of this study lies in its use of highly detailed data. While previous studies often relied on larger analytical units due to difficulties in data collection, we utilized approximately 3 million individual business data points to develop a more accurate and systematic deep learning-based spatial pattern prediction model. This approach provides a robust quantitative foundation for future policy-making. Please see section 2.4. Literature gaps.

2. The manuscript lists seven independent variables used in the LSTM model. However, the rationale for selecting these specific variables is insufficiently explained. While the literature is cited, it is not clear why these particular variables were chosen over others. Additionally, the manuscript should discuss whether other potentially relevant variables were considered and why they were excluded. For example, factors such as local economic indicators, consumer behavior data, or external shocks (e.g., economic crises, pandemics) could significantly impact survival rates.

The author’s answer

We considered but could not include variables such as industry type, number of employees, region, and economic growth rate due to the model limited specifically to the Seoul city and data limitations. Including these variables in future research could enhance the accuracy of the model.

As the reviewer pointed, we did not provide sufficient explanation of the dependent and independent variables used in the model. We added an explanation of why the cutoff years were set to 5 years for survival rate prediction in this study. Please see line 170-175. We also updated a paragraph explaining the variables we considered but could not include in the model, along with the reasons for their exclusion. Please see line 176-184. I appreciate your suggestion.

3. It would be beneficial to include a discussion comparing LSTM with other potential models, such as AR, MA, ARIMA, GRU (Gated Recurrent Unit).

The author’s answer

We inserted a paragraph comparing the LSTM model with other models we considered. Please see line 207-217 and 442-443. I appreciate your suggestion.

4. Given that the classification of commercial districts can significantly influence the results and interpretations, it would be beneficial to conduct a sensitivity analysis to determine how changes in the classification thresholds might affect the outcomes. For instance, what happens if the thresholds are adjusted to mean ±1 standard deviation? Does the distribution of commercial districts change significantly, and how does this impact the subsequent analysis? Including a sensitivity analysis would provide more confidence in the robustness of the classification method.

The author’s answer

As the reviewer pointed out, we also carefully considered how to set the classification thresholds and determined that using mean ± 0.5 standard deviation (SD) was the most appropriate approach.

The first reason is data-driven. Analyzing the basic statistics of business density per pixel shows that the mean is 34.09, the standard deviation is 43.82, and the distribution is skewed to the right (Fig 1). Since the standard deviation is larger than the mean, setting the threshold at mean ± 1 SD results in mean - 1 SD being less than zero, which would eliminate the low-density category entirely. To address this, we considered thresholds of mean ± 0.4 SD, mean ± 0.5 SD, and mean ± 0.6 SD. Among these, the pixel counts for the three density types were the most balanced when using mean ± 0.5 SD(Table 1). In comparison, mean ± 0.4 SD resulted in nearly half of the pixels being classified as low-density, while mean ± 0.6 SD resulted in nearly half being classified as medium-density.

**Fig 1. Histogram of the number of businesses per pixel.**

|  | low density | medium density | high density |
| --- | --- | --- | --- |
| mean+-0.4sd | 1927 (47.8%) | 1187 (29.4%) | 920 (22.8%) |
| mean+-0.5sd | 1668 (41.3%) | 1525 (37.8%) | 841 (20.8%) |
| mean+-0.6sd | 1329 (32.9%) | 1966 (48.7%) | 739 (18.3%) |

**Table 1. The pixel counts for the three density types.**

The second reason is alignment with real-world conditions. When using mean ± 0.5 SD as the threshold, the classification most accurately reflects the reality of commercial districts in Seoul. For instance, Fig 2 shows the developed commercial district areas provided by the Seoul Metropolitan Government.

In Seoul, commercial districts are classified based on density into alley commercial districts and developed commercial districts to facilitate statistical analysis and policy-making for commercial area support. Among these, developed commercial districts are defined as areas with higher density and more active commercial activities. When comparing the high-density commercial districts classified using the mean ± 0.5 SD threshold in this study with the developed commercial districts identified by the Seoul Metropolitan Government, the spatial distributions of the two are highly similar. This alignment supports the validity of the density classification method used in this study and its reflection of real-world conditions.

|   **A. High-density commercial districts** |   **B. Developed commercial districts.** (Source: Seoul Metropolitan Government) |
| --- | --- |

**Fig 2. Classifying commercial districts by business density.**

We summarized the content and incorporated it into the manuscript along with the map. Please see line 359-363 and Fig 8. I appreciate your suggestion.

5. The manuscript provides a general description of how different business densities are distributed across Seoul (e.g., high-density districts are major commercial areas). However, the spatial distribution of these density classifications is not deeply analyzed in relation to the urban geography of Seoul. The manuscript should explore how these classifications relate to existing urban structures, such as zoning regulations, transportation networks, and residential patterns. For example, are high-density commercial districts primarily located near major transportation hubs or within specific urban zones? Understanding these spatial relationships would add depth to the analysis and provide more meaningful insights into urban planning.

6. The manuscript does not address whether the classification of business densities is stable over time or if there are significant temporal variations. For instance, a district classified as medium-density in one time period might transition to high-density in another period due to urban development or economic shifts.

The author’s answer

We inserted a section explaining the spatial distribution of density types in relation to Seoul's urban geography and examining their temporal stability. Please see lines 340-363 and Fig 8. I appreciate your suggestion.

7. Survival rate dynamics capture changes over time, but the manuscript does not discuss the potential for lag effects, where changes in survival rates may not immediately reflect broader economic or social trends. For instance, a commercial district might experience a decline in survival rates due to a delayed response to economic downturns or shifts in consumer behavior.

The author’s answer

As the reviewer suggested, changes in small business survival rates do not immediately reflect economic or social trends. We inserted this limitation of the study. Please see line 487-489. I appreciate your suggestion.

8. Were the data normalized or standardized?

The author’s answer

We did not apply normalization or standardization to the data used for training the LSTM model. There are two potential approaches for normalizing the data: first, normalizing across the group of 4,034 pixels for each time interval, and second, normalizing across the three-time intervals for each pixel. The first approach would replace the pixel values with relative values compared to other pixels within Seoul, which would make it challenging to reflect absolute changes when analyzing the data over time. On the other hand, the second approach is limited to having only three-time intervals for each pixel, which results in a group size too small for effective normalization. In this study, the model achieved a high level of accuracy despite not applying normalization. However, if more extended time-series data becomes available, normalization processes, as suggested by the reviewer, could further improve the accuracy of the model results.

9. Is there any missing data? How were missing values handled? Any imputation methods used?

The author’s answer

In this study, pixels without commercial facilities, for which data could not be generated, were excluded from the analysis. We updated the manuscript to include this information. Please see line 144-146. I appreciate your suggestion.
