# Supplementary material for: Classification of commercial districts based on predicting the survival rate of food service market in Seoul
Source: PLoS One. 2025 Jul 10;20(7):e0326307. doi: 10.1371/journal.pone.0326307 (PMC12244476; doi:10.1371/journal.pone.0326307)
Supplement: Attachment — Submitted filename: Response_to_Reviewers_auresp_2.docx [file pone.0326307.s004.docx]

**Responses to Editor**

Thank you for your valuable comments. The recommendations and comments definitely improved the quality of this paper. Below are the major changes made during the revision process.

Please review your reference list to ensure that it is complete and correct. If you have cited papers that have been retracted, please include the rationale for doing so in the manuscript text, or remove these references and replace them with relevant current references. Any changes to the reference list should be mentioned in the rebuttal letter that accompanies your revised manuscript. If you need to cite a retracted article, indicate the article’s retracted status in the References list and also include a citation and full reference for the retraction notice.

The author’s answer

We have reviewed the reference list and revised it to comply with the journal’s formatting guidelines. There are no retracted articles cited in this manuscript. However, we found a few formatting inconsistencies in the reference list, which have now been corrected. We appreciate your suggestion.

**Responses to Reviewer 1**

Thank you for your valuable comments. The recommendations and comments definitely improved the quality of this paper. Below are the major changes made during the revision process.

Thank you for your hard work. From the perspective of the methodology, I think you have done a very good job.

The author’s answer

We sincerely appreciate your encouraging remarks. Your accurate and constructive comments have contributed significantly to improving the quality of our research.

**Responses to Reviewer 2**

Thank you for your valuable comments. The recommendations and comments definitely improved the quality of this paper. Below are the major changes made during the revision process.

Given the comprehensiveness, techniques, and detailed description of the conducted research, I would recommend the paper to be published (after addressing 2 very minor items identified below)

The author’s answer

We sincerely appreciate your thoughtful review and recommendation for publication.

1. In the paragraph that describing the data/features used in the modeling, authors mentioned that they were relying on information based from "One hundred industries closely tied to daily life", however, I'm not sure if I'm seeing corresponding reference here, could we please add this information.

The author’s answer

As the reviewer suggested, we have added a citation for the "One Hundred Industries Closely Tied to Daily Life" and included additional details to provide a clearer description of this classification. Please see lines 192-194. We appreciate your suggestion.

2. I'm not sure if I missed it, but I don't think I'm seeing Figure 3. LSTM conceptual diagram in both the main content nor supplement materials.

The author’s answer

As per the journal's submission guidelines, we did not embed the figures directly in the manuscript but submitted them as separate files.
For your convenience, we have also included Figure 3 (LSTM conceptual diagram) in this response letter.

**Fig 3. LSTM conceptual diagram.**

**Responses to Reviewer 3**

Thank you for your valuable comments. The recommendations and comments definitely improved the quality of this paper. Below are the major changes made during the revision process.

1) given the dynamic nature of commercial districts, it would be interesting to discuss the potential for using the LSTM model for dynamic prediction or real-time predictions. For example, could the model be integrated into a dashboard that provides up-to-date survival rate predictions for different districts?

(Taylor JM, Yu M, Sandler HM. Individualized Predictions of Disease Progression Following Radiation Therapy for Prostate Cancer. Journal of Clinical Oncology 2005; 23(4): 816–825.)

The author’s answer

As the reviewer pointed out, integrating the LSTM model into a real-time dashboard for the dynamic prediction of commercial district survival rates is feasible and holds substantial utility for practice and policy. Given the LSTM architecture’s proficiency in capturing patterns within sequential and time-dependent data, the predictive model can be continuously updated by incorporating live data streams, thus enhancing the responsiveness and relevance of survival forecasts. We have expanded the discussion to include broader applications of the LSTM-based predictive model. Please see lines 456-458. We appreciate your suggestion.

2) Has cross validation been used for prediction accuracy?

The author’s answer

In this study, conventional cross-validation techniques were not employed in the analysis of the Long Short-Term Memory (LSTM) model. This methodological decision was informed by the intrinsic properties of time series data and the architectural characteristics of the LSTM model, for the following reasons.

First, time series data are inherently sequential and exhibit autocorrelation among observations. This temporal dependency constitutes a critical component of the information structure that models must learn. Traditional K-fold cross-validation, which randomly partitions the dataset, disrupts this temporal order, thereby undermining the validity of the performance evaluation in time-dependent contexts. Accordingly, evaluation methods that preserve the sequential integrity of the data are more appropriate for time series forecasting tasks.

Second, to assess predictive performance while maintaining temporal consistency, we adopted a time-based hold-out validation strategy. In this approach, earlier observations were used to train the model, while subsequent observations were reserved for validation. This chronological split closely mirrors real-world forecasting scenarios and allows for a more meaningful evaluation of the model's predictive capabilities in practice.

Third, deep learning architectures such as LSTM, which fall under the broader category of recurrent neural networks, are computationally intensive and require considerable time and resources for training. Implementing repeated training procedures, as required by traditional cross-validation, would impose substantial computational burdens. Given that the primary objective of this study was not to conduct model benchmarking but rather to uncover and interpret temporal patterns within specific time series clusters, a more resource-efficient and contextually appropriate validation approach was warranted.

For these reasons, conventional cross-validation was deemed unsuitable for the present study. Instead, an alternative evaluation method—tailored to the characteristics of time series data and aligned with the study’s analytical objectives—was adopted.

As the reviewer pointed out, cross-validation is necessary to enhance the reliability of the model. In our next study, we will utilize various accuracy validation techniques, such as Nested cross-validation and Hold-out cross-validation, to improve the model’s accuracy through cross-validation. We appreciate your suggestion.
